# Design and rationale of a multi-center, pragmatic, open-label randomized trial of antimicrobial therapy – the study of clinical efficacy of antimicrobial therapy strategy using pragmatic design in Idiopathic Pulmonary Fibrosis (CleanUP-IPF) clinical trial

**DOI:** 10.1186/s12931-020-1326-1

**Published:** 2020-03-12

**Authors:** Kevin J. Anstrom, Imre Noth, Kevin R. Flaherty, Rex H. Edwards, Joan Albright, Amanda Baucom, Maria Brooks, Allan B. Clark, Emily S. Clausen, Michael T. Durheim, Dong-Yun Kim, Jerry Kirchner, Justin M. Oldham, Laurie D. Snyder, Andrew M. Wilson, Stephen R. Wisniewski, Eric Yow, Fernando J. Martinez, For the CleanUP-IPF Study Team, Joan Albright, Kevin J. Anstrom, Emily S. Clausen, Joanna Cole, Dahlia Cowhig, Coleen Crespo, Michael Durheim, Jerry Kirchner, Heather Kuehn, Jay Rao, Laurie D. Snyder, Qinghong Yang, Eric Yow

**Affiliations:** 1 Duke Clinical Research Institute, Duke University, Durham, North Carolina USA; 2 Division of Pulmonary Medicine, University of Virginia, Charlottesville, Virginia USA; 3 Division of Pulmonary & Critical Care Medicine, University of Michigan Health System, Ann Arbor, MI USA; 4 Pulmonary Fibrosis Foundation, Chicago, IL USA; 5 Graduate School of Public Health, University of Pittsburgh, Pittsburgh, PA USA; 6 Norwich Medical School, University of East Anglia, Norwich, UK; 7 Division of Pulmonary and Critical Care Medicine, University of Pennsylvania, Philadelphia, PA USA; 8 Department of Respiratory Medicine, Oslo University Hospital – Rikshospitalet, Oslo, Norway; 9 National Heart, Lung and Blood Institute, National Institutes of Health, Bethesda, MD USA; 10 UC Davis, Pulmonary, Critical Care, and Sleep Medicine, Davis, California USA; 11 Division of Pulmonary Medicine, Weill-Cornell Medical Center, Cornell University, New York, NY USA

**Keywords:** Idiopathic pulmonary fibrosis, Pragmatic clinical trial, Doxycycline, Co-trimoxazole

## Abstract

**Abstract:**

Compelling data have linked disease progression in patients with idiopathic pulmonary fibrosis (IPF) with lung dysbiosis and the resulting dysregulated local and systemic immune response. Moreover, prior therapeutic trials have suggested improved outcomes in these patients treated with either sulfamethoxazole/ trimethoprim or doxycycline. These trials have been limited by methodological concerns. This trial addresses the primary hypothesis that long-term treatment with antimicrobial therapy increases the time-to-event endpoint of respiratory hospitalization or all-cause mortality compared to usual care treatment in patients with IPF. We invoke numerous innovative features to achieve this goal, including: 1) utilizing a pragmatic randomized trial design; 2) collecting targeted biological samples to allow future exploration of ‘personalized’ therapy; and 3) developing a strong partnership between the NHLBI, a broad range of investigators, industry, and philanthropic organizations. The trial will randomize approximately 500 individuals in a 1:1 ratio to either antimicrobial therapy or usual care. The site principal investigator will declare their preferred initial antimicrobial treatment strategy (trimethoprim 160 mg/ sulfamethoxazole 800 mg twice a day plus folic acid 5 mg daily or doxycycline 100 mg once daily if body weight is < 50 kg or 100 mg twice daily if ≥50 kg) for the participant prior to randomization. Participants randomized to antimicrobial therapy will receive a voucher to help cover the additional prescription drug costs. Additionally, those participants will have 4–5 scheduled blood draws over the initial 24 months of therapy for safety monitoring. Blood sampling for DNA sequencing and genome wide transcriptomics will be collected before therapy. Blood sampling for transcriptomics and oral and fecal swabs for determination of the microbiome communities will be collected before and after study completion. As a pragmatic study, participants in both treatment arms will have limited in-person visits with the enrolling clinical center. Visits are limited to assessments of lung function and other clinical parameters at time points prior to randomization and at months 12, 24, and 36. All participants will be followed until the study completion for the assessment of clinical endpoints related to hospitalization and mortality events.

**Trial Registration:**

ClinicalTrials.gov identifier NCT02759120.

## Background

IPF is a chronic, fibrotic, and progressive interstitial lung disease characterized by the histopathologic pattern of usual interstitial pneumonia in the absence of an identifiable cause or association. Disease progression is highly heterogeneous with a median survival of approximately 3–5 years following diagnosis. Furthermore, the increasing rate of mortality and hospitalization related to the disease suggests that the prevalence is increasing [1]. Studies of pirfenidone and nintedanib have shown consistent beneficial effects in forced vital capacity and led to approval of both agents by the U.S. Food and Drug Administration [1–3]. However, both agents demonstrated inconsistent benefits on clinical endpoints, may be difficult to tolerate, and are expensive. As a result, there remains an unmet clinical need for effective and low cost treatment strategies to improve the quality-of-life and clinical outcomes in patients with IPF.

Here we describe the design and rationale for CleanUP-IPF clinical trial. In particular, the pragmatic nature of the study represents the first IPF study that may demonstrate a significant treatment effect for a clinical endpoint and offers a model to identify effective treatment strategies for rare diseases.

## Methods

### What is the rationale for the antimicrobial therapies?

Compelling data have linked disease progression with lung dysbiosis and the resulting local and systemic immune response in IPF patients [4–8]. Murine data support the impact of lung microbes on increased fibrotic response [9, 10]. In other chronic disorders, antimicrobial therapy has been suggested to favorably alter the lung microbial community [11]. This trial utilizes a pragmatic approach with antimicrobial agents that have been suggested to have a similar effect in IPF patients. The use of two potentially effective therapies minimizes potential risk while increasing the number of patients that can be treated with such innovative therapy.

*What is the rationale for using co-trimoxazole?* An initial randomized trial of 20 patients with advanced fibrotic lung disease showed favorable improved exercise capacity and symptom scores in the participants assigned to co-trimoxazole [12]. Following these results, a UK National Institute for Health Research funded study called TIPAC randomized 180 patients with interstitial lung disease to co-trimoxazole or placebo [13]. The primary endpoint was forced vital capacity. An as-treated analysis suggested favorable results for quality-of-life and all-cause mortality. Based on these findings, the investigators hypothesized that a larger study with better treatment adherence could prove that co-trimoxazole is a cheap and effective therapy for IPF. A limitation of the TIPAC study was the lack of significant findings using the intention-to-treat analyses. A further clinical trial, EME-TIPAC, is underway to replicate this study in a larger study population [14].

*What is the rationale for using doxycycline?* A prior single-center study examined 6 patients with IPF treated with long-term doxycycline [15]. Patients were treated for a mean of 303 days with assessments of body mass index, 6-min walk test, St. George Respiratory Questionnaire, FVC, and several biomarkers. Patients were included if they signed informed consent documents, had an IPF diagnosis from a pulmonologist and radiologist (major and minor criteria according to the ATS-ERS guidelines of 2001) age 30–70 years, and FVC percent predicted > 40%. Briefly, patients were excluded if they had a contraindication to doxycycline or a recent exacerbation of IPF among other reasons. Patients received 100 mg of doxycycline once daily if body weight was < 50 kg and 100 mg of doxycycline twice daily if body weight was > 50 kg. A key study endpoint was inhibition of MMP activity in BAL fluid after at least 6 months of therapy. The study results include large but not statistically significant changes in 6-min walk distance (141 ft, *p* = 0.110) and FVC percent predicted (6.3%, *p* = 0.311). [Appendix Table 3] Additionally, the study found large and statistically significant changes in St. George Respiratory Questionnaire, MMP9 activity, MMP3 activity, MMP9 expression, TIMP-1 expression, and VEGF expression. In spite of these consistent differences, this study has a number of limitations. A separate open label study in a small number of patients treated with a mean of 531 days of doxycycline experienced improvement clinically, physiologically and radiologically [16]. These small, single center studies did not have a proper control group and had a relatively unstructured protocol. Nevertheless, these case series suggest that doxycycline has the potential to be an effective treatment for IPF given the high responsiveness of the anti-MMPs activity.

### Pulmonary Trials Cooperative

In 2014, the National Institutes of Health (NIH) issued a pair of funding opportunity announcements for applications to create the Pulmonary Trials Cooperative (PTC). https://grants.nih.gov/grants/guide/rfa-files/RFA-HL-15-015.html and https://grants.nih.gov/grants/guide/rfa-files/RFA-HL-15-016.html. One announcement called for U01 applications to serve as the Protocol Leadership Group (PLG) and the other announcement called for a Network Management Core (NEMO) to serve as the clinical coordinating body for the PTC. The PTC was designed to conduct multiple simple, pragmatic Phase II and III studies to evaluate the potential benefits of new and existing treatment strategies. The primary responsibility of the NEMO, which is coordinated by investigators at the University of Pittsburgh, is to facilitate the trials conducted by the PTC. The primary responsibility of the PLGs is to develop a protocol and provide the necessary resources to support the conduct and data analyses for that project. The NEMO recruits and activates a number of clinical sites to identify and enroll patients depending on the study protocol. In general, the role of the clinical sites is to enroll participants, deliver the study intervention, complete study visits (in-person and phone calls), conduct procedures as defined in the study protocol, aid in data interpretation and participate in manuscript generation. Currently, the PTC is conducting four randomized controlled trials – three in patients with chronic obstructive pulmonary disease [INSIGHT-COPD (NCT02634268), LEEP (NCT02696564), and RETHINC (NCT02867761)] and one in patients with IPF, CleanUP-IPF (http://www.pulmonarytrials.org/). The CleanUP-IPF PLG is led by investigators from Weill Cornell Medicine, University of Virginia, and the Duke Clinical Research Institute.

### CleanUP-IPF study overview

Participants will be randomized to one of two strategies – usual care or usual care plus anti-microbial therapy in a 1:1 allocation ratio. Prior to randomization, eligible participants and their physician will declare a preference for the co-trimoxazole or the doxycycline stratum. It is expected that the majority of participants will be in the co-trimoxazole stratum. Once participants are randomized to usual care or usual care plus anti-microbial therapy, their follow-up schedule will vary based on their assigned therapy (i.e. usual care, co-trimoxazole, or doxycycline). Participants in the anti-microbial strategy will receive a voucher to help cover the costs associated with the study medications. Compared with standard clinical trials in patients with IPF, the in-person follow-up visits will be infrequent (e.g. similar to usual care at most US clinical centers). A robust protocol has been implemented to track the participants for potential safety issues. Suspected clinical events of interest, specifically hospitalizations and acute worsening, will be reviewed by an independent adjudication committee. The study will be reviewed by an independent NIH-appointed Data and Safety Monitoring Board (DSMB). It is expected that all patients will be followed until a common end date based on the study progress.

### Key design elements

As described earlier, the FOA requested proposals for simple, pragmatic Phase II and III clinical trials. There is considerable variability in the definitions and interpretations of pragmatic clinical trials. Often, pragmatic trials are designed to capitalize on previously captured data (e.g. electronic health records), information collected from participants during their usual activities (e.g. patient reported outcomes), and a patient-centric design [17, 18]. For many researchers, the ADAPTABLE clinical trial assessing the benefits and effectiveness of two different aspirin dosing strategies is considered a highly pragmatic study [19, 20]. Similarly, there is considerable debate about the nature and utility of large simple trials [21]. Some argue that there should be many more clinical trials that enroll large number of participants and result in minimal burden for patients and enrolling sites. Others note that such trials fit a niche; however, they generally will fail to serve a purpose given regulatory and logistical constraints [22].

In an attempt to answer a clinically important question, the study investigators designed a very streamlined clinical trial with an existing therapy in a highly generalizable population. This approach was in response to the increasingly complicated and burdensome clinical trial environment [23, 24]. After funding was awarded, the study team added several refinements to the study protocol that made the study more flexible and safer. The PRECIS-2 tool is a commonly used tool to assess pragmatism of clinical research studies [25–29]. The tool was developed from the input of dozens of clinical trialists and measures 9 different aspects of the clinical trial – eligibility criteria, recruitment, setting, study organization, flexibility of delivery, flexibility of adherence, follow-up, primary outcome, and primary analysis. Each domain is scored from 1 (very explanatory) to 5 (very pragmatic). Table 1 shows the PRECIS-2 domains and the CleanUP-IPF investigator’s opinions on the pragmatism for each of them. In the opinion of the investigators, all of the 9 domains scored between moderately pragmatic and very pragmatic.

**Table 1:** PRECIS-2 Domains and the CleanUP-IPF Design

PRECIS-2 Domains [Loudon BMJ 2015]	PRECIS-2 Score for CleanUP-IPF
*1. Eligibility—*To what extent are the participants in the trial similar to those who would receive this intervention if it was part of usual care?	Median Investigator Score* – 5 Very Pragmatic All patients who would receive the treatment if the drugs in CleanUP-IPF are found to be effective have been enrolled. No additional procedures have been required of patients to enroll in the study. The design allows physicians to identify and diagnosis patients according to their usual practice. The PTC has attempted to identify a group of clinics that are more generalizable than prior IPF studies which relied primarily on large academic medical centers. The exclusions are tightly aligned with the subset of patients who are unlikely to receive the treatment if the trial is positive (e.g. those with contra-indications).
*2. Recruitment—*How much extra effort is made to recruit participants over and above what would be used in the usual care setting to engage with patients?	**Median Investigator Score – 5 Very Pragmatic** Patients in CleanUP-IPF are primarily identified from routine clinic visits and little effort is made to identify patients using electronic health records or mailings. The NIH and PTC have invested very limited amounts to support the enrolling sites. Payments to enrolling sites are strictly tied to enrollment and data collection (i.e. there are no infrastructure payments). Patients enrolled in CleanUP-IPF receive a study drug voucher which serves to partially cover the cost of study medications. Additionally patients enrolled at certain sites receive reimbursement for certain study related activities such as parking and gas mileage.
*3. Setting*—How different are the settings of the trial from the usual care setting?	**Median Investigator Score – 4.5 Rather Pragmatic** CleanUP-IPF is being conducted in a single country; however, the expectation would be that the treatment(s) are applied regardless of the country of residence for the patient. The PTC is making an effort to identify a representative set of sites to enroll patients. The total number of enrolling sites is expected to reach approximately 30–40. The majority of sites are tied to major academic medical centers. This set of sites reasonably matches the sites that are expected to treat this fairly rare and difficult to diagnose disease. The PTC is working to ensure that the sex, racial, and ethnicity characteristics of enrolled populations closely match the broader population with the disease. Most of the study sites identify and enroll patients at the clinics where these patients are seen in usual practice.
*4. Organization*—How different are the resources, provider expertise, and the organization of care delivery in the intervention arm of the trial from those available in usual care?	**Median Investigator Score – 4 Rather Pragmatic** The CleanUP-IPF study has attempted to structure the study to closely mimic the ultimate delivery of the treatment, if and when, it is moved to usual care. Certain design features including the use of a voucher system to reimburse care do not match the intended delivery. The study investigators and coordinators have received ample training from the PTC but that training was mostly designed to improve the proper execution of the clinical research. The study investigators did not require any additional study training or years of experience to be recruited into the PTC site list. The ultimate delivery of the antimicrobial therapy would not require additional health care resources or staff.
*5. Flexibility (delivery)—*How different is the flexibility in how the intervention is delivered and the flexibility anticipated in usual care?	**Median Investigator Score – 4 Rather Pragmatic** The CleanUP-IPF study does employ a highly protocol driven assessment of safety for patients randomized to the antimicrobial treatment strategy. However, there are no programs in place to improve compliance with of the enrolling physicians. The timing of the intervention is not tightly defined and can be applied at any point during the chronic phase of the disease. There are no restrictions placed on other potential therapies used to treat IPF. Restrictions and monitoring of other therapies are driven by safety concerns.
*6. Flexibility (adherence)—*How different is the flexibility in how participants are monitored and encouraged to adhere to the intervention from the flexibility anticipated in usual care?	**Median Investigator Score – 4 Rather Pragmatic** The eligibility criteria did not place any restrictions on the ability of participants to be complaint during the trial. The study does not withdraw any patients from the trial for the lack of compliance to study procedures. The study team does not explicitly meet with enrolling sites to discuss issues related to adherence to study drug. The flexibility for patients enrolled is very high with allowances to switch to a different study drug if there are issues with the assigned therapy.
*7. Follow-up*—How different is the intensity of measurement and follow-up of participants in the trial from the typical follow-up in usual care?	**Median Investigator Score – 4.5 Rather Pragmatic** The CleanUP-IPF follow-up schedule was closely tied to the follow-up for IPF patients in usual care. There are addition assessments at the 12 and 24 months that are included to provide the key data for secondary endpoints. The intervention arm has a few additional follow-up telephone calls and blood draws to assess the patient for any potential safety issues. Sites are encouraged to use data from the electronic health record to use for assessments related to lung function when possible. There are no follow-up visits that are triggered based on potential endpoint events. Most participants enrolled in the study are also contributing data to several ancillary studies which require additional blood and stool samples.
*8. Primary outcome—*To what extent is the trial’s primary outcome directly relevant to participants?	**Median Investigator Score – 5 Very Pragmatic** The key question that CleanUP-IPF is attempted to address is whether the use of antimicrobial therapy reduces mortality and respiratory related hospitalizations. All-cause mortality has been identified as the most important endpoint for patients with IPF. Similarly, the need for acute care in the form of hospitalizations is an outcome that patients would prefer to avoid. Traditional phase II and III trials in IPF have used biomarkers related to lung function or functional assessments such as 6-min walk distance as the primary endpoint [30]. The rationale for this decision is usually tied to feasibility concerns related to time-to-event studies with clinical endpoints. It is our understanding that CleanUP-IPF will be one of the first IPF trials to use a clinical endpoint as the primary outcome. Similarly, the sample size is believed to be the largest IPF trial conducted only in the US. A slight weakness of the primary outcome is the inclusion of the non-fatal respiratory hospitalization component [31]. The trial will use a central adjudication process for the mortality and hospitalization events which lowers the pragmatism. The time horizon for CleanUP-IPF with a maximum follow-up of 3 years is highly pragmatic.
*9. Primary analysis—*To what extent are all data included in the analysis of the primary outcome?	**Median Investigator Score – 5 Very Pragmatic** CleanUP-IPF uses a superiority design and the primary analysis population is based on all randomized patients. There are no special allowances for redefining the population for issues related to imperfect adherence or changes in the eligibility criteria that are identified after randomization. The use of all-cause mortality in the primary endpoint means that individuals with deaths that are unrelated to the disease or treatment are still included in the analysis. Similarly, the use of the respiratory hospitalization component means that patients with lung transplantation are included in the primary analysis. The primary analysis will use a covariate adjusted model but those covariates are expected to be obtained in 100% of patients prior to randomization. It is expected that the primary outcome will have nearly complete data at the time of the final study visits.

*Scores are based on survey responses from 14 CleanUP-IPF clinical sites

### Protocol specifics

#### Study objective

The primary objective of the study is to compare usual care vs. usual care plus antimicrobial therapy (co-trimoxazole or doxycycline) on clinical outcomes in patients diagnosed with IPF. The hypothesis is that reducing harmful microbial impact with antimicrobial therapy will reduce the risk of non-elective, respiratory hospitalization or death in patients with IPF. A total of 30–40 U.S. clinical centers are expected to enroll a total of 500 participants.

#### Eligibility

The detailed inclusion and exclusion criteria are enumerated in Table 2. There are a total of three inclusion criteria, only one of which requires any clinical information. Another ongoing clinical trial, EME-TIPAC, studying a similar hypothesis at approximately 40 U.K. sites used a more explanatory approach including the use of a placebo-controlled design [14]. The studies are very similar in terms of the inclusion criteria but clearly differ when examining the exclusion criteria. For CleanUP-IPF, the exclusions only prohibit those with contraindications to the study interventions.

**Table 2:** Comparison of CleanUP-IPF with EME-TIPAC eligibility criteria

Inclusion Criteria	
CleanUP-IPF (NCT 02759120)	**EME-TIPAC** **(ISRCTN 17464641)**
1. ≥ 40 years of age 2. Diagnosed with IPF by enrolling investigator 3. Signed informed consent	1. Age greater than or equal to 40 years 2. A diagnosis of IPF based on multi-disciplinary consensus according to the latest international guidelines. 3. Patients may receive oral prednisolone up to a dose of 10 mg per day, anti-oxidant therapy, pirfenidone or other licensed medication for IPF e.g. nintedanib. Patients should be on a stable treatment regimen for at least 4 weeks to ensure baseline values are representative. 4. MRC dyspnea score of greater than 1. 5. Able to provide informed consent
Exclusion Criteria	Exclusion Criteria (as of January 7, 2019)*
1. Received antimicrobial therapy in the past 30 days for treatment purposes (antibiotic prophylaxis for procedures do not meet criteria, nor do antivirals) 2. Contraindicated for antibiotic therapy 3. Pregnant or anticipate becoming pregnant 4. Use of an investigational study agent for IPF therapy within the past 30 days, or an IV infusion with a half-life of four (4) weeks 5. Concomitant immunosuppression with azathioprine, mycophenolate, cyclophosphamide, or cyclosporine.	1. FVC > 75% predicted. 2. A recognized significant co-existing respiratory disease, defined as a respiratory condition that exhibits a greater clinical effect on respiratory symptoms and disease progression than IPF as determined by the principal investigator. 3. Patients with airways disease defined as forced expiratory volume in 1 s (FEV1)/FVC < 60% 4. A self-reported respiratory tract infection within 4 weeks of screening defined as two or more of cough, sputum or breathlessness and requiring antimicrobial therapy. 5. Significant medical, surgical or psychiatric disease that in the opinion of the patient’s attending physician would affect subject safety or influence the study outcome including liver (Serum transaminase > 3 x upper limit of normal (ULN), Bilirubin > 2 x ULN) and renal failure (creatinine clearance < 30 ml/min). 6. Patients receiving recognized immunosuppressant medication (except prednisolone above) including azathioprine and mycophenolate mofetil. 7. Female subjects must be of non-childbearing potential, defined as follows: postmenopausal females who have had at least 12 months of spontaneous amenorrhea or 6 months of spontaneous amenorrhea with serum FSH > 40mIU/ml or females who have had a hysterectomy or bilateral oophorectomy at least 6 weeks prior to enrollment. 8. Allergy or intolerance to trimethoprim or sulphonamides or their combination. 9. Untreated folate or B12 deficiency. 10. Known glucose-6-phosphate dehydrogenase (G6PD) deficiency or G6PD deficiency measured at screening in males of African, Asian or Mediterranean descent. 11. Receipt of an investigational drug or biological agent within the 4 weeks prior to study entry or 5 times the half-life if longer. 12. Receipt of short course antibiotic therapy for respiratory and other infections within 4 weeks of screening. 13. Patients receiving long term (defined as > 1 month of therapy) prophylactic antibiotic treatment will not be eligible as this may have an impact on lung microbiota. Such patients may enroll in the EME-TIPAC trial, if this is supported by their clinician, after a ‘wash-out period’ of 3 months. 14. Serum Potassium greater than 5.0 mmol/l due to the potentially increased risk of hyperkalemia in patients taking co-trimoxazole in combination with potassium sparing diuretics (including angiotensin converting enzyme inhibitors or angiotensin receptor blockers)

*The study eligibility criteria are taken verbatim from the official trial registration (http://www.isrctn.com/ISRCTN17464641)

#### Interventions

**Fig. 1:**
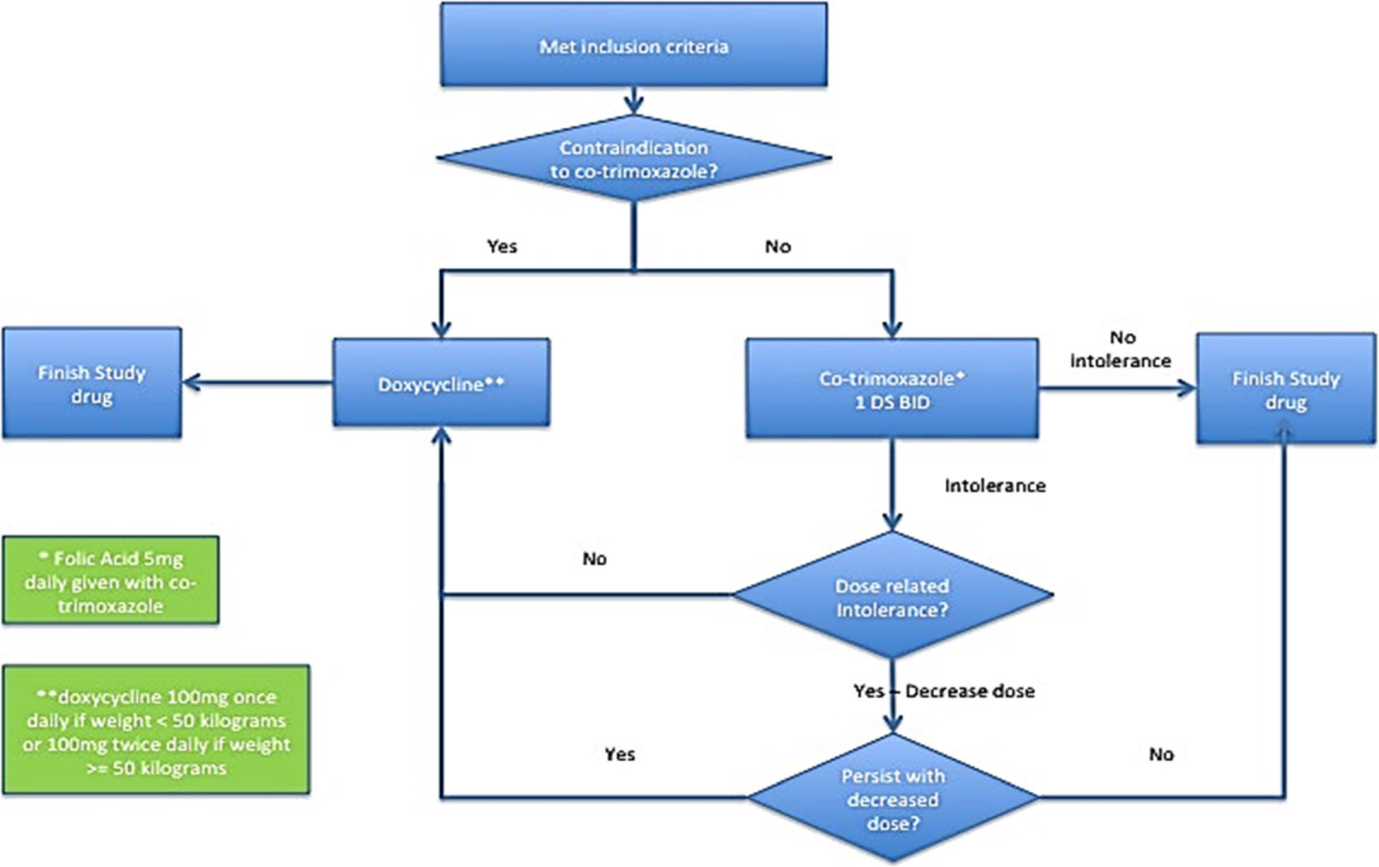
Flow diagram for participants randomized to antimicrobial therapy

Participants randomized to antimicrobial therapy will be treated with trimethoprim 160 mg/sulfamethoxazole 800 mg (double strength co-trimoxazole) twice a day plus folic acid 5 mg daily unless there is a contraindication to this therapy. The addition of folate administration was employed to minimize the risk of leukopenia associated with inhibition of folic acid metabolism by trimethoprim [32]; folic acid replacement has been used successfully with chronic use of this antimicrobial agent in HIV patients and patients with interstitial lung disease [13, 33]. If the participant develops an intolerance to co-trimoxazole, the dosage can be decreased to trimethoprim 160 mg/sulfamethoxazole 800 mg (one double strength co-trimoxazole) three times weekly plus folic acid 5 mg daily. If intolerance continues with co-trimoxazole, then the antimicrobial agent can be changed to doxycycline (without folic acid). See Fig. 1 for the flow diagram for participants randomized to antimicrobial therapy. Participants in the doxycycline cohort who are randomized to usual care plus antimicrobial therapy will be treated with doxycycline (without folic acid) with a weight-based dosing (100 mg once daily if body weight is < 50 kg and 100 mg twice daily if ≥50 kg).

#### Pre-randomization evaluations

Prior to randomization, the study coordinator will collect the following information:

- Patient characteristics (sex, race, ethnicity, age, height, weight)
- Information on how IPF diagnosis was made
- Co-morbidities and details on patient history of gastroesophageal reflux disease (GERD)
- Physical exam findings
- Current concomitant medications
- Urine dipstick pregnancy test
- Evaluation of renal function
- Evaluation of potassium level
- Evaluation of leukocyte count and platelet count in recipients randomized to co-trimoxazole

In addition, the following procedures will be performed prior to randomization unless recent clinically indicated tests are available:

- Spirometry and DLCO
- Quality of life questionnaires
- Buccal and fecal sample collection
- Blood draw for genotype and gene expression
- Chemistry panel and liver function tests
- Complete Blood Count

#### Duration of intervention

After randomization, participants assigned to the antimicrobial arm will be given a prescription drug voucher from Trialcard to help defray the cost of study drug. Participants will have minimal in-person visits over the course of the 36-month study but those visits depend on the assigned study arm. Participants assigned to the usual care arm have scheduled in-clinic visits at 12 and 24 months. Participants in the antimicrobial arm have additional visits at 1 week, 3 months, and 6 months to monitor safety related to the study drugs.

#### Diagnosis

The diagnosis of participants will be highly pragmatic. The diagnosis of IPF within the trial will match the processes used to diagnose the disease based on international guidelines [34, 35]. The study will collect information on how IPF diagnosis was made using an IPF Diagnosis Checklist.

#### Safety related concerns & safety reviews

The safety testing in this study is based on prior experience with these antimicrobial agents in other settings [33, 36–45]. Participants are encouraged to follow the assigned treatment strategy for the study duration; however, in all cases the participant’s safety based on the clinical judgment of the treating physician will take priority over the specific treatment assignment. There is the potential of adverse cardiovascular events secondary to co-trimoxazole therapy; this is felt to possibly reflect a trimethoprim drug interaction resulting in hyperkalemia [38, 41]. Review of prior literature suggests that the major risk factors for trimethoprim related hyperkalemia include higher trimethoprim dose, arenal insufficiency with hypoaldosteronism, potassium altering medications, and age [40]. Our inclusion/exclusion criteria should mitigate this risk as well as monitoring for hyperkalemia early after the introduction of co-trimoxazole therapy [40].

#### Primary Endpoints & Endpoint Adjudication

The primary endpoint of this study will be the time to first non-elective, respiratory hospitalization or all-cause mortality. The significance of respiratory hospitalization as a potential trial endpoint in IPF has been demonstrated in several studies [46]. In pooled data from the IPF Clinical Research Network (IPFnet) clinical trials, both non-elective hospitalization and disease progression as defined by a 10% decrease in FVC occurred frequently across strata of baseline physiologic impairment. Both of these events were associated with subsequent time to death from any cause. After adjustment for gender, age, and baseline lung function, the risk of all-cause mortality during trial follow-up was nearly six-fold higher among patients who had a non-elective hospitalization of respiratory cause early during the trial, compared with those who had not (hazard ratio [HR] 5.97, 95% confidence interval [CI] 1.81, 19.74). By contrast, non-respiratory hospitalizations were not associated with subsequent risk of mortality. These findings build upon earlier observations both in clinical trials and in clinical practice [47, 48]. As such, non-elective respiratory hospitalization appears to be the optimal clinical intermediate marker for long-term mortality in IPF. This evidence has been incorporated in CleanUP-IPF, including the use of an adjudication group based on IPFnet experience [49]. The CleanUP-IPF event adjudication process is designed to be both efficient and accurate, incorporating the assessments of both the local site investigator and an independent central adjudication to confirm the clinical cause of a hospitalization or mortality event.

#### Secondary endpoints

A number of clinical events, quality-of-life, and lung function measures have been identified as secondary endpoints. These include:

- Time to death from any cause
- Time to first non-elective, respiratory hospitalization
- Time to first non-elective, all-cause hospitalization
- Total number of non-elective respiratory hospitalizations
- Total number of non-elective all-cause hospitalizations
- Change in FVC from randomization to 12 months
- Change in DLCO from randomization to 12 months
- Total number of respiratory infections
- UCSD-Shortness of Breath Questionnaire at 12 months
- Fatigue Severity Scale score at 12 months [50]
- Leicester Cough Questionnaire score at 12 months [51]
- EQ-5D score and SF-12 score at 12 months
- ICEpop CAPability measure for Older people score at 12 months [52, 53]

#### Safety endpoints

The electronic data collection forms will collect a targeted set adverse events of special interest such as arrhythmia, diarrhea, hyperkalemia, rash, and vomiting.

#### General statistical considerations

In this unblinded trial, all participants will be randomized to treatment in a 1:1 allocation ratio using a simple randomization scheme within the electronic data collection system. It was the belief of the investigators that blinding would add substantial additional complexity without commensurate incremental benefit related to testing the primary hypothesis of a treatment strategy trial. Means, standard deviations, medians, 25th and 75th percentiles will be presented for continuous variables; the number and frequency of patients in each category will be presented for nominal variables. Statistical tests with a two-sided *p* value < 0.05 will be considered statistically significant, unless otherwise stated. Analyses will be performed using SAS software (SAS Institute, Inc., Cary, NC).

#### Analysis of the primary endpoint

Detailed description of the plan for statistical analysis of each endpoint will be produced in a separate Statistical Analysis Plan. The primary analysis will be based on intention to treat. Crossovers (e.g. drop-in and drop-out) will be tracked and an alternate analysis cohort will be developed based on these data. Participants receiving lung transplantation during the course of follow-up will be censored for all endpoints at the time of transplantation.

The statistical comparison of the two randomized arms with respect to the primary endpoint will be a time-to-event analysis, and therefore will be based on the time from randomization to first non-elective, respiratory hospitalization or death from any cause. The Cox proportional hazards regression model will be the primary tool to analyze and assess outcome differences between the two treatment arms. The Cox model will include an indicator variable for treatment group, age, sex, baseline DLCO, baseline FVC, use of N-Acetylcysteine at enrollment, indicator variables for the use of nintedanib or pirfenidone at enrollment, and choice of antimicrobial agent prior to randomization. Hazard ratios and 95% confidence intervals will summarize the differences between treatment arms. Kaplan-Meier estimates will be used to display event rates by treatment group.

For the primary analysis, participants who are event-free (i.e. subjects without any respiratory hospitalization or death event at the time of analysis) will be censored at their last visit or lung transplantation. The censoring mechanism is assumed to be non-informative. Supportive analyses will be performed to assess the impact of a potential informative censoring.

#### Sample size and power calculations

Based on IPFnet data, it is anticipated that the event rate in the placebo arm will be highly dependent on the proportion of patients enrolled at the different gender, age, and lung physiology (GAP) index scores [31, 54]. Given the availability of two U.S. Food and Drug Administration (FDA)-approved drugs for IPF, it is our belief that the study population will be heavily weighted toward GAP index scores of 3. In Appendix Table 4, the statistical power is determined for designs enrolling 500 participants with usual care group events rates varying from 24 to 36% and (12-month) treatment effects varying from 30 to 35%. In general, the proposed design provides adequate power except when the 12-month standard-of-care group event rate is below 24% and the reduction in events is less than 30%. We plan to enroll 500 patients window with a minimum of 12 months of follow-up on all patients. Appendix Table 5 shows the required number of endpoint events to have adequate power across varying hazard ratios.

#### Data and safety monitoring board

The NIH-appointed DSMB includes individuals with pertinent expertise in IPF, clinical trials, ethics and biostatistics. The DSMB will advise the PLG and the NIH regarding the continuing safety of current participants and those yet to be recruited. The DSMB will meet approximately 2 times per year to review safety and overall study progress until the end of the study.

#### DSMB monitoring plan

Prior to each meeting, the data coordinating center at Duke Clinical Research Institute will conduct any requested statistical analyses and prepare a summary report along with the following information: patient enrollment reports, rates of compliance with the assigned testing strategy, frequency of protocol violations, and description of serious adverse events. There will be one planned interim review for efficacy. The efficacy review will focus on the composite endpoint of respiratory hospitalization or all-cause death and should occur once 300 enrolled subjects have been followed for 12 months. The Lan-DeMets alpha spending function with O’Brien-Fleming type boundaries will be used for the interim analysis.

## Discussion

### Endpoint issues in IPF studies

There has been considerable debate in the IPF clinical research world about the appropriate endpoint for Phase III clinical trials [30, 31, 55–58]. To date, both FDA-approved drugs (nintedanib and pirfenidone), have used FVC as the primary endpoint. As a result, the majority of Phase II and III clinical trials in IPF have used the measure of lung function as the primary endpoint. Recently, there has been considerable work on quality of life and symptoms (including cough and reflux) [59]. Furthermore, several groups have pooled clinical trial databases to examine treatment effects of drugs on clinical endpoints including mortality and respiratory hospitalizations [60, 61]. The CleanUP-IPF trial has been designed to have a composite clinical primary endpoint as part of the Prospective Open Label Blinded Endpoint (PROBE) design [62].

### Public-private partnership

The parent structure for the trial utilizes the NHLBI sponsored PTC. A large network of clinical centers ranging from community-based centers to tertiary institutions is conducting the study**.** Financial support for this study includes contributions from three additional organizations: Three Lakes Partners, IPF Foundation, and Veracyte, Inc. Three Lakes Partners is a venture philanthropy whose mission is to accelerate the development of promising technologies for IPF (https://threelakespartners.org/). The mission of the IPF Foundation is to advocate and fundraise for the most promising research to accelerate IPF cures (https://ipffoundation.org). Veracyte, Inc. is a pioneer in genomic diagnostics (https://www.veracyte.com) that has developed a genomic classifier that facilitates the diagnosis of usual interstitial pneumonia and potentially IPF [63].

In summary, the CleanUP-IPF study has several potentially transformative elements. The pragmatic design is reducing the participant burden and allowing for a large enough sample size to evaluate clinical endpoints. Additionally, the highly flexible design will allow for mechanistic studies, collection of biological samples, and pooling of the study database with the EME-TIPAC study. Finally, the study leverages a comprehensive private-public partnership including the NIH, a broad range of investigative institutions, philanthropic organizations, and industry.

## Data Availability

Not applicable.

## Appendix 1

**Table 3:** Doxycycline study - comparisons of enrollment and follow-up assessments*

Endpoint	N	Enrollment Mean (SD)	Follow-up Mean (SD)	Paired T-test *p*-value
Body Mass Index (kg/m^2^)	6	25.41 (4.41)	26.07 (4.45)	0.080
6 Minute Walk Test (feet)	5	1142 (159)	1283 (194)	0.110
St. George’s Respiratory Questionnaire – total score	6	50.90 (8.38)	18.40 (6.39)	0.002
FVC percent predicted (%)	6	61.38 (10.65)	67.67 (14.39)	0.311
MMP9 activity	6	6.19 (2.04)	2.59 (0.66)	0.006
MMP3 activity	6	9.03 (2.02)	4.83 (3.54)	0.041
MMP9 expression	6	3.39 (1.06)	1.45 (0.41)	0.004
TIMP-1 expression	6	5.28 (1.56)	2.72 (0.67)	0.018
VEGF expression	6	9.03 (2.02)	4.83 (3.54)	0.041

*Data are taken from [15]. Activities levels are determined from Western Blot. See [15] for more details

## Appendix 2

**Table 4:** Statistical Power Assuming a Sample Size of 500 Randomized Patients

Standard-of-care event rate*	Antimicrobial therapy strategy event rate*	One-year Event Rate Reduction	Power
24%	16.8%	30%	78%
30%	21.0%	30%	87%
36%	25.2%	30%	93%
24%	16.0%	33.3%	86%
30%	20.0%	33.3%	93%
36%	24.0%	33.3%	97%
24%	15.6%	35%	89%
30%	19.5%	35%	95%
36%	23.4%	35%	98%

*12-month event rates. Calculations assume a 2-sided Type-I error rate of 0.05. The minimum follow-up is planned to be 12 months and the maximum follow-up is 42 months. Drop-out rates are assumed to be approximately 2% per year. Power calculations were based on a log-rank test with assumed event rates were exponentially distributed. Calculations were computing using nQuery 7.0 software

## Appendix 3

**Table 5:** Required number of primary endpoint events

	HR = 0.50	HR = 0.55	HR = 0.60	HR = 0.65	HR = 0.70	HR = 0.75
80% power	65	88	120	169	247	379
85% power	75	100	138	194	282	434
90% power	87	118	161	226	330	508

Calculations performed using nQuery 7.0 and assume a 0.05 type I error rate (two-sided) with 1:1 randomization

## Abbreviations

CI: Confidence interval
DLCO: Diffusing capacity for carbon monoxide
DSMB: Data and safety monitoring board
FDA: U. S. food and drug administration
FVC: Forced vital capacity
GAP: Gender, age, and lung physiology
HR: Hazard ratio
IPF: Idiopathic pulmonary fibrosis
IPFnet: IPF clinical research network
NEMO: Network management core
NIH: National institutes of health
PLG: Protocol leadership group
PRECIS-2: PRagmatic explanatory continuum indicator summary-2
PROBE: Prospective open label blinded endpoint
PTC: Pulmonary Trials Cooperative
